# Pseudoleukocytosis secondary to hepatitis C-associated cryoglobulinemia: a case report

**DOI:** 10.1186/1752-1947-3-91

**Published:** 2009-11-02

**Authors:** Abdallah Geara, Badiaa El-Imad, Walid Baz, Marcel Odaimi, Suzanne El-Sayegh

**Affiliations:** 1 Department of Internal Medicine, Staten Island University Hospital, 475 Seaview Avenue, Staten Island, NY 10305, USA; 2 Department of Hematology and Oncology, Staten Island University Hospital, 475 Seaview Avenue, Staten Island, NY 10305, USA; 3 Department of Nephrology, Staten Island University Hospital, 475 Seaview Avenue, Staten Island, NY 10305, USA

## Abstract

**Introduction:**

Laboratory tests play a central role in assessing a patient and orienting the diagnostic evaluation. We report a case where the discrepancy between the manual and automatic cell count gave a hint to the final diagnosis.

**Case presentation:**

A 55-year-old Caucasian man, known to have hepatitis C, was admitted with acute respiratory failure secondary to acute pulmonary edema and diffuse petechial rash of the lower extremities for the previous 2 months. The initial laboratory tests showed acute renal failure (creatinine of 2.6 mg/dL). During his hospital stay, the patient had a fluctuating white blood cell count with a recorded value of 96,000 cells/mL. On a peripheral smear, the blood cell count was in the normal range. The acute renal failure was secondary to membranoproliferative glomerulonephritis secondary to essential mixed cryoglobulinemia diagnosed by biopsy. The complete blood count values, performed by Beckman/Coulter GenS, were falsely high due to precipitation of plasma cryoglobulins at room temperature. This spurious leukocytosis was previously described in several case reports, but values as high as 96,000 cells/mL were never reported.

**Conclusion:**

The presence of cryoglobulins in the blood creates a clinical challenge for the interpretation of several laboratory tests. Pseudoleukocytosis secondary to cryoglobulinemia has been observed in several reported cases with white blood cell counts up to 54,000 cells/mL at room temperature and 85,600 cells/mL at 4°C. If the cryoglobulin precipitates rapidly, aggregated cryoglobulin particles may be interpreted as blood cells. We report the first patient with pseudoleukocytosis secondary to hepatitis C cryoglobulinemia with a spurious leukocytosis of 96,000 cells/mL at room temperature. Other laboratory tests could also be affected: underestimation of true erythrocyte sedimentation rate, pseudothrombocytosis and pseudolymphocytosis. The precipitation can remove the hepatitis C virus and the antibody of cryoglobulins from serum leading to a false negative result. Any discrepancy between the automated and manual white blood cell count should lead to the suspicion of cryoglobulinemia in the clinical setting.

## Introduction

Laboratory tests play a central role in assessing a patient and orienting the diagnostic evaluation. In some clinical situations, the results of laboratory tests could be affected by the method used to perform the test (that is to say, pseudohyperkalemia in patients with a high platelet count [1], pseudohypoglycemia in polycytosis [2]). We report a case where the discrepancy between the manual and automatic cell count gave a hint to the final diagnosis.

## Case presentation

A 55-year-old American Caucasian man presented to the emergency department with acute respiratory failure secondary to acute pulmonary edema and diffuse petechial rash. The rash had been present for the previous 8 months, starting as a macular rash in both lower extremities and progressing to involve the trunk and upper extremities. In addition, for the last 2 months, he had complained of shortness of breath that limited his activity and which was progressively deteriorating. He was known to have hepatitis C, diagnosed when he was 1 year old, not treated and an anxiety disorder being treated with a benzodiazepine.

Upon admission to the emergency department, the patient was intubated and ventilated. Cardiac evaluation revealed a left ventricular ejection fraction of 30%. Initial laboratory tests showed acute renal failure (creatinine of 2.6 mg/dL), low serum albumin (2.9 g/dL) and protein (4.7 g/dL) and a normocytic anemia (hematocrit of 26.3%). Urinalysis showed microscopic hematuria (30-40 red blood cells/high power field (HPF)) with 3-6 coarse granular casts/low power field (LPF), and significant proteinuria (1600 mg of proteins/1 g of creatinine).

**Figure 1:**
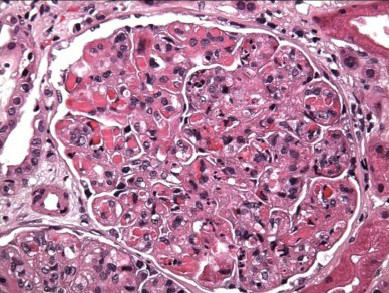
**Renal biopsy: diffuse endocapillary proliferative and exudative glomerulonephritis with membranoproliferative features and numerous intracapillary protein thrombi**.

**Figure 2:**
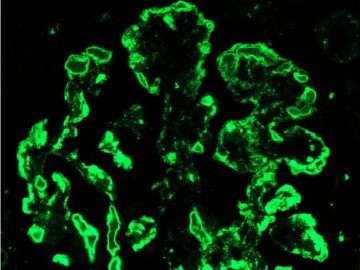
**Immunofluorescence findings of intense glomerular capillary wall and intraluminal staining in the distribution of the protein thrombi for IgM, C3 and C1q**.

Investigation for the etiology of the acute renal failure was compatible with acute nephritic syndrome with low complement levels (C3 = 54 mg/dL (normal: 79-152); C4 = 2.7 mg/dL (normal: 16-38)). Rheumatoid factor was 150 IU/mL (normal: 0-20), erythrocyte sedimentation rate (ESR) was 1, and anti-nuclear antibodies (ANA) and cryoglobulin collected at room temperature were negative. A kidney biopsy showed a membranoproliferative glomerulonephritis secondary to essential mixed cryoglobulinemia type II (Figure 1 and Figure 2). Hepatitis C viral ribonucleic acid (RNA) was 1,350,000 IU/mL, and the genotype was 1a. The patient was started on exchange plasmapheresis and prednisone, and his kidney function improved. Treatment of hepatitis C was deferred until stabilization of the renal failure.

During his hospital stay, the patient had fluctuations of his white blood cell count (WBC). On admission and during his stay in the intensive care unit (ICU), his WBC count was in the range of 8000 to 12,000 cells/mL; after his transfer to the ward, his WBC counts varied between 24,000 and 96,000 cells/mL. On the peripheral smear, the blood cell count was in the normal range. The complete blood count values, performed by Beckman/Coulter GenS, were falsely high due to the precipitation of plasma cryoglobulins at room temperature. After treatment with plasmapheresis, the WBC counts tested on several occasions were in the normal range. The leukocytosis in our patient was secondary to the cryoglobulins.

## Discussion

Cryoglobulins are serum proteins that precipitate in the cold. Usually these cryoglobulins are either immunoglobulins or a mixture of immunoglobulins and complements. Although cryoglobulinemia is asymptomatic in most cases, it can lead to immune complex tissue deposition causing cryoglobulinemic vasculitis. In 1974, Brouet et al. [3] classified cryoglobulinemia into three types (I, II and III), type II and III usually associated with hepatitis C virus (HCV) infection. In chronic HCV infection, cryoglobulins are found in 55-90% of patients of whom 2-15% develop cryoglobulinemic vasculitis [4]. The clinical manifestations are mostly cutaneous and renal. The skin lesions are usually palpable purpura of the lower extremities. The cryoglobulin could deposit in the renal glomeruli leading to proteinuria, nephritic syndrome secondary to membranoproliferative glomerulonephritis, Fanconi's syndrome, acute and chronic renal failure. The presence of cryoglobulins in the blood can create a clinical challenge for the interpretation of several laboratory tests.

First, the detection of false negative cryoglobulinemia is highly probable if the test is not performed in the appropriate way: the blood should be drawn preferably in a warm container, the tube should be maintained at 37°C during transport to the laboratory and during the centrifugation process, and then refrigerated at 4°C for 48 to 72 hours. Since some of these steps were not performed, the initial negative cryoglobulin test in our patient was a false negative result. We were able to detect cryoglobulins in the serum of this patient after repeating the test and the kidney biopsy was positive for cryoglobulins.

Second, cryoglobulins can affect the result of the complete blood cell count performed by an automated counter. Pseudoleukocytosis secondary to cryoglobulinemia has been observed in several reported cases with a WBC count as high as 54,000 cells/mL at room temperature [5] and 85,600 cells/mL at 4°C [6]. Haeney [7] studied the total leukocyte count at different temperatures in two patients with cryoglobulinemia, and found an increase in the WBC count as the temperature was lowered: although the WBC count was normal at 37°C, it rose to as high as 20,000 cells/mL after 1 hour at 4°C. In electronic cell counters, the individual cells flow through an aperture where there is an electrical current. The cell produces resistance to the electrical current. The increased resistance is sensed by the instrument and registered as a cell. In cryoglobulinemia, when the blood cell count is processed at temperatures lower than 37°C, the cryoglobulin precipitates at low temperature and increases the resistance to the electrical current. This can lead to falsely elevated blood cell counts and platelets in cryoglobulinemia. In order to avoid unnecessary investigations in patients with vasculitis and suspiciously elevated blood counts, blood cell counts should be performed after the blood has been heated to 37°C [8].

Although, pseudoleukocytosis secondary to cryoglobulinemia has been described in several case reports, this is the first patient with pseudoleukocytosis with proven hepatitis C-associated cryoglobulinemia. In addition, the highest value recorded for leukocytosis at room temperature was 54,000 cells/mL; our patient had a WBC count as high as 96,000 cells/mL at room temperature.

Other laboratory tests can also be affected. The erythrocyte sedimentation readings at 20°C and 12°C underestimate the true ESR [7]. Pseudothrombocytosis [9] and pseudolymphocytosis [10] were even reported with doubling of the platelet counts.

Finally, the presence of cryoglobulin in the blood could raise a challenge in identifying the underlying infectious agent associated with this vasculitis. The precipitation of cryoglobulins from serum can remove the hepatitis C virus and the antibody. The serum can test negative for HCV even by polymerase chain reaction, despite the presence of persistent viremia [11]. In these cases, performing the test on the collected precipitate could give accurate results.

## Conclusion

Cryoglobulin-induced laboratory artifacts could be a leading element to suspect cryoglobulinemia in several previous case reports [8]. The highest reported pseudoleukocytosis secondary to cryoglobulinemia was 54,000 cells/mL at room temperature. Our patient had a falsely high leucocyte count of 96,000 cells/mL secondary to aggregation of cryoglobins at room temperature. The etiologies of spurious leucocytosis are either platelet aggregation or cryoglobulinemia. Any discrepancy between the automated and manual WBC count should lead to the suspicion of cryoglobulinemia in the clinical setting.

## Abbreviations

HPF: high power field; LPF: low power field; ESR: erythrocyte sedimentation rate; ANA: anti-nuclear antibody; RNA: ribonucleic acid; WBC: white blood cells; ICU: intensive care unit; HCV: hepatitis C virus

## Consent

Written informed consent was obtained from the patient for publication of this case report and any accompanying images. A copy of the written consent is available for review by the Editor-in-Chief of this journal.

## Competing interests

The authors declare that they have no competing interests.

## Authors' contributions

AG and WB were involved in the care of the patient, in establishing the diagnosis and reviewing the literature. BI and AG collaborated in writing the article. BI also contributed in reviewing and analyzing the literature. MO and SS provided the final review of the article and the analysis of the peripheral smear and the renal biopsy.
